# UDP-glucuronic acid availability underlies sex difference in renal expression of nonsulfated Human Natural Killer-1 (HNK-1) glycans

**DOI:** 10.1371/journal.pone.0335730

**Published:** 2025-11-13

**Authors:** Tomoko Onishi, Hajime Okayama, Hitomi Kitano, Katsuaki Higashi, Naoki Nakagawa, Motohiro Nonaka, Shogo Oka, Jyoji Morise

**Affiliations:** Human Health Sciences, Graduate School of Medicine, Kyoto University, Kyoto, Japan; Meijo University, JAPAN

## Abstract

Nonsulfated Human Natural Killer-1 (nsHNK-1) glycan is a unique trisaccharide structure terminating in glucuronic acid and synthesized by glucuronyltransferase GlcAT-S. This glycan is specifically expressed in the mouse kidney, particularly in proximal tubules and the thick ascending limb of Henle’s loop. Renal nsHNK-1 glycan exhibits an age-dependent increase, indicating that its expression is strictly regulated. However, previous studies have primarily focused on male kidneys, leaving potential sex differences unexplored. In this study, we found that renal nsHNK-1 glycan expression is significantly higher in female mice compared with male mice. Notably, no sex differences were observed in the expression of N-acetyllactosamine structures, the substrate for glucuronic acid modification, or in GlcAT-S expression levels. Moreover, analysis of knockout mice for GlcAT-P, an isoform of GlcAT-S, confirmed that GlcAT-P also does not contribute to the sex differences in nsHNK-1 glycan expression. These findings prompted us to investigate the intracellular availability of uridine diphosphate glucuronic acid (UDP-GlcA), the donor substrate for GlcAT-S, as a possible contributor to sex-specific renal nsHNK-1 glycan expression. To investigate this hypothesis, we developed a quantitative ELISA to measure intracellular UDP-GlcA levels. *In vivo*, wild-type female mice exhibited lower renal UDP-GlcA levels compared with males. However, this difference was abolished in GlcAT-S knockout mice, suggesting enhanced UDP-GlcA consumption in female mice. In HK-2 cells, derived from human proximal tubular epithelium, cultivation under high-glucose conditions elevated intracellular UDP-GlcA, resulting in increased nsHNK-1 glycan expression. Conversely, stimulation of UDP-GlcA consumption via glucuronidation using 4-methylumbelliferone suppressed the high-glucose-induced increase in nsHNK-1 glycan expression levels. Taken together, these findings identify UDP-GlcA availability as a key determinant of nsHNK-1 glycan biosynthesis in the kidney, highlighting a novel regulatory mechanism that contributes to sex-specific glycan expression.

## Introduction

The Human Natural Killer-1 (HNK-1) glycan is composed of a trisaccharide structure containing sulfated glucuronic acid at the nonreducing terminus, which is biosynthesized by two glucuronyltransferases, GlcAT-P (*B3GAT1*) and GlcAT-S (*B3GAT2*), and the sulfotransferase HNK-1ST (*CHST10*) [1]. This glycan is specifically expressed in the nervous system, where it plays a crucial role in regulating synaptic plasticity and contributes to spatial memory and learning [2,3]. Thus, HNK-1 glycan is a functional structure that governs higher brain functions. In contrast, the mouse kidney specifically expresses a nonsulfated form of the HNK-1 glycan (nsHNK-1) because HNK-1ST expression is absent [4]. Furthermore, GlcAT-P is barely expressed in the mouse kidney, resulting in the biosynthesis of terminal glucuronic acid modifications primarily by GlcAT-S. Mice with a GlcAT-S gene knockout (SKO) exhibit an almost complete absence of nsHNK-1 glycan expression in the kidney [5,6], indicating that GlcAT-S serves as the rate-limiting enzyme in nsHNK-1 glycan biosynthesis. Although the functional roles of HNK-1 glycan in the nervous system have been elucidated, the role of nsHNK-1 glycan in the kidney remains largely unexplored. The expression pattern of nsHNK-1 glycan has been analyzed in the mouse kidney to gain insight into its physiological relevance. nsHNK-1 glycan displays region-specific expression in the nephron, with restriction to proximal tubules and the thick ascending limb of Henle’s loop [4]. The major carrier proteins for nsHNK-1 glycan are membrane-bound metalloproteases meprin A subunit alpha (MEP1A) and aminopeptidase N (APN), both of which are localized to proximal tubules [7,8], with nsHNK-1 being present on their N-glycans [9]. Additionally, nsHNK-1 glycan expression in the mouse kidney increases with age [9]. In α-klotho mice, a model of premature aging, renal nsHNK-1 glycan expression is significantly higher compared with wild-type (WT) mice of the same age [9]. Given that renal function generally declines with age, these findings suggest a potential link between nsHNK-1 glycan expression and age-related changes in renal physiology.

Uridine diphosphate glucuronic acid (UDP-GlcA) serves as the primary donor substrate for GlcAT-S-mediated transfer of glucuronic acid to N-acetyllactosamine (LacNAc) structures during the biosynthesis of nsHNK-1 glycan. UDP-GlcA is synthesized in the cytoplasm via the oxidation of UDP-glucose by UDP-glucose dehydrogenase [10], forming a crucial metabolic branch of glucose utilization. In addition to its role in glycosylation, UDP-GlcA is consumed in glucuronidation, a detoxification pathway mediated by UDP-glucuronosyltransferases (UGTs) that conjugate glucuronic acid to a wide range of substrates [11]. Given these competing demands, intracellular levels of UDP-GlcA are expected to be strictly regulated and may vary depending on metabolic conditions, tissue type, and biological context, including sex. However, whether differences in UDP-GlcA availability contribute to the regulation of nsHNK-1 glycan expression has not been addressed previously.

Sex differences influence gene expression and molecular profiles in various tissues, including the kidney [12]. Recent transcriptome analyses have revealed that genes involved in glycosylation are differentially expressed between males and females in several human organs [13,14]. In the kidney, sex-dependent differences have been reported in glycosaminoglycan content and glycosphingolipid composition [15–18]. Additionally, RNA-sequencing focusing on proximal tubules has shown sex-biased expression of multiple glycosyltransferases [19,20]. These observations suggest that sex-specific glycosylation patterns play a role in maintaining normal renal function in males and females. However, whether such sex differences also apply to nsHNK-1 glycan expression in the kidney remains unknown.

In this study, we first found that nsHNK-1 glycan expression is significantly higher in female mice than in male mice. However, the expression levels of GlcAT-S and the abundance of LacNAc structures did not differ between sexes, indicating that these factors do not explain the mechanism of sex difference in nsHNK-1 glycan expression. These observations led us to hypothesize that differences in the availability of UDP-GlcA may play a regulatory role. To investigate this possibility, we developed a quantitative ELISA-based assay to measure intracellular UDP-GlcA concentrations *in vivo* and examined the effects of modulating UDP-GlcA metabolism on nsHNK-1 glycan expression *in vitro*. Our findings reveal a critical role for UDP-GlcA in regulating nsHNK-1 glycan biosynthesis, providing new insights into the metabolic and sex-specific regulation of renal glycan expression.

## Materials and methods

### Animal experiments

All animal procedures were conducted in accordance with the Fundamental Guidelines for Proper Conduct of Animal Experiments and Related Activities in Academic Research Institutions under the jurisdiction of the Ministry of Education, Culture, Sports, Science and Technology of Japan. Protocols were approved by the Animal Experimentation Committee of Kyoto University (MedKyo: 21504, 22093, 23078, 24111, 25125). Unless otherwise specified, all mice used in this study were of the C57BL/6J strain. Generation and genotyping of SKO and GlcAT-P gene knockout (PKO) mice have been described previously [2,5]. To collect tissues, euthanasia was performed either by cervical dislocation under deep isoflurane anesthesia or by CO_2_ inhalation, in accordance with institutional guidelines. For perfusion fixation experiment, mice were anesthetized by intraperitoneal injection of a mixed anesthetic consisting of medetomidine hydrochloride (0.3 mg/kg), midazolam (4 mg/kg), and butorphanol tartrate (5 mg/kg) to alleviate suffering.

### Preparation of renal membrane fractions

Adult mouse kidneys were homogenized in five volumes of ice-cold homogenization buffer (0.25 M sucrose, 10 mM Tris-HCl [pH 7.4], 1 mM EDTA, and protease inhibitor cocktail; Cat#25955−11, Nacalai Tesque, Kyoto, Japan). Homogenates were centrifuged at 1,000 × g for 10 min to remove nuclei, and the supernatant was further centrifuged at 105,000 × g for 1 h. The resulting pellet was resuspended in 1% SDS and used as the renal membrane fraction.

### Western and lectin blotting

Membrane fractions were separated by SDS-PAGE (7% or 10%) and transferred onto nitrocellulose membranes. Precision Plus Protein™ All Blue Prestained Standards (Cat#1610373, Bio-Rad, Hercules, CA, USA) were used as molecular weight markers. Membranes were blocked with 5% skim milk in phosphate-buffered saline (PBS) containing 0.05% Tween-20 (PBST), incubated with primary antibodies (1 µg/mL), followed by horseradish peroxidase (HRP)-conjugated secondary antibodies (1 µg/mL). For lectin blotting, membranes were blocked with Blocking One (Cat#03953–95, Nacalai Tesque) and incubated with HRP- or biotin-conjugated lectins diluted in 5% Blocking One in PBST. When biotin-conjugated lectins were used, HRP labeling was carried out according to the manufacturer’s instructions provided with VECSTAIN ABC Kit (Cat#PK-4000, Vector Laboratories, Newark, CA, USA). Signals were developed using SuperSignal™ West Pico Chemiluminescent Substrate (Cat#34580, Thermo Fisher Scientific, Waltham, MA, USA) and visualized with a LuminoGraph II system (ATTO, Tokyo, Japan). Band intensities were quantified using ImageJ software. Primary antibodies: M6749 monoclonal antibody (mAb; kindly provided by Dr. H. Tanaka, Kumamoto University [21]), anti-β-actin mAb (Cat#M177-3, MBL, Tokyo, Japan), anti-APN mAb (Cat#ab108310, Abcam, Cambridge, UK), and anti-MEP1A polyclonal antibody (pAb; Cat#PA5–81444, Thermo Fisher Scientific). Secondary antibodies: HRP-conjugated anti-mouse IgM pAb (Cat#62–6820), HRP-conjugated anti-mouse IgG pAb (Cat#62–6520), and HRP-conjugated anti-rabbit IgG pAb (Cat#65–6120), purchased from Thermo Fisher Scientific, respectively. Lectins: HRP-conjugated RCA120 lectin (from Lectin Set-HRP 1; Cat#J4S1, J-OIL MILLS, Tokyo, Japan), Biotin-conjugated MAM lectin (specific for α2–3-linked sialic acids; Cat#J210, J-OIL MILLS), and SiaFind α2–6 Specific Reagent Biotinylated Kit (specific for α2–6-linked sialic acids; Cat# SK2601B, Lectenz Bio, Athens, GA, USA). A polyclonal antibody against GlcAT-S (anti-GlcAT-S pAb) was generated using the same method previously employed for GlcAT-P [22], by immunizing a rabbit with the recombinant catalytic region of human GlcAT-S expressed and purified from *Escherichia coli*.

### Real-time quantitative PCR

Total RNA from kidneys was extracted using TRIzol (Cat#15596018, Thermo Fisher Scientific), and cDNA synthesized from 2 µg RNA using oligo(dT) primer (Cat#3805, Takara Bio, Shiga, Japan) and SuperScript™ III Reverse Transcriptase (Cat#18080093, Thermo Fisher Scientific). qPCR was performed using StepOnePlus system (Thermo Fisher Scientific) and TB Green Premix Ex Taq II (Cat#RR820A, Takara Bio) according to the manufacture procedure. Primer pairs were used as follows: *B4galt1*, 5’-agcaactcgactatggcatc-3’, 5’-tccatcggaatgaggtccac-3’; *Mgat4a*, 5’-acaaaccggtcaacgtggag-3’, 5’-ccctctgcaacaccatactc-3’; *Mgat4b*, 5’-aagcctcaagacgtaccagc-3’, 5’-gcagcacctccacagaagtg-3’; *Mgat5*, 5’-cccttgtgtatggcaaagtg-3’, 5’-gtcacgtccactgagaatgc-3’; *B3gat2* (mouse GlcAT-S), 5’-gacgatgacaacacgtacag-3’, 5’-taccaacggacgttcatagc-3’; *B3gat1* (mouse GlcAT-P), 5’-gctcatcttgcagcgaagtc-3’, 5’-ccctcattgaccagcactgg-3’; *Ugdh*, 5’-agtgagctacagaccattgg-3’, 5’-aacttcggaatctcaccagg-3’; *Uxs1*, 5’-ccactcacagtctatggctc-3’, 5’-cacttccactaccaacaagg-3’; *Gapdh*, 5’-acttcaacagcaactcccac-3’, 5’-atgtaggccatgaggtccac-3’. Each primer was used at 0.4 µM. Reactions were run in duplicate for three biological replicates. Relative expression was calculated by the ΔΔCt method, using *Gapdh* as an internal control.

### Immunofluorescence staining

Mice were deeply anesthetized and perfused transcardially with PBS, followed by 4% paraformaldehyde in PBS. Then, kidneys were immersed in 30% sucrose, sectioned at 40 µm using a microtome, and blocked with 3% bovine serum albumin (BSA) in PBS (BSA/PBS). Sections were incubated with M6749 mAb, anti-megalin pAb (Cat#ab76969, Abcam), and anti-MEP1A pAb (2.5 µg/mL in 3% BSA/PBS, respectively) for 2 h, followed by FITC-conjugated anti-mouse IgM pAb (1:400 in 3% BSA/PBS; Cat#31992, Thermo Fisher Scientific) or Alexa Fluor™ 488-conjugated anti-rabbit IgG pAb (1:400 in 3% BSA/PBS; Cat#A21206, Thermo Fisher Scientific) for 1 h. All steps were conducted at room temperature. Images were acquired using a FLUOVIEW FV1000 imaging system (Olympus, Tokyo, Japan).

### UDP-GlcA extraction

UDP-GlcA was extracted from mouse kidney tissues or cultured cells based on a protocol from a previous report [23]. Briefly, ~ 25 mg of tissues or cells were homogenized in 500 µL of 60% methanol. After additional sonication, they were mixed with 225 µL chloroform. After centrifugation at 18,000 × g for 3 min at 4 °C, the upper aqueous layer was collected, dried in a vacuum evaporator, and reconstituted in ultrapure water.

### ELISA for UDP-GlcA quantification

UDP-GlcA levels were quantified using a modified ELISA method based on a previous report with slight modification, originally developed for the quantification of UDP-N-acetylglucosamine [23]. Briefly, microplates were coated with 0.01% poly- L-lysine (Cat#P2636, Sigma-Aldrich, St. Louis, MO, USA) for 4–6 h at room temperature, followed by 0.5 µg asialorosomucoid (ASOR) overnight at 4 °C. ASOR is a glycoprotein in which terminal sialic acids on N-glycans have been removed, thereby exposing LacNAc structures that serve as acceptor substrates for GlcA transfer. In this study, ASOR was prepared according to a previously reported method [24]. A reaction mixture containing 0.1 M MES (pH 6.5), 0.2% Nonidet P-40, 10 mM MnCl₂, 2.5 mM adenosine triphosphate (ATP), and 10 µg/mL recombinant GlcAT-P (prepared according to a previous procedure [25]), along with samples and/or UDP-GlcA standards (Cat#217−00953, FUJIFILM Wako, Osaka, Japan), was added and incubated at 37 °C for 1 h. Note that recombinant GlcAT-P was used in this assay, as the binding to ASOR was not clearly observed with GlcAT-S and had been reliably analyzed only with GlcAT-P in the previous surface plasmon resonance experiments [26]. Plates were blocked with 1% BSA in Tris-buffered saline (TBS) and sequentially incubated with M6749 mAb (1 µg/mL in 1% BSA/TBS containing 0.1% Tween-20) and HRP-conjugated anti-mouse IgM pAb (1:5000, in 1% BSA/TBS containing 0.1% Tween-20). The reaction was developed using SureBlue™ TMB 1-Component Microwell Peroxidase Substrate (Cat#5120−0081, LGC Clinical Diagnostics, Milford, MA, USA), terminated with 1 M HCl, and absorbance at 450 nm was measured using an Infinite M200 plate reader (Tecan, Zürich, Switzerland). UDP-GlcA concentrations were determined based on absorbance values calibrated against a standard curve, and normalized to the wet weight of tissue or cells used in each assay, yielding values expressed in pmol/mg.

### Expression plasmids and cell culture

The rat GlcAT-S expression plasmid, pEF-BOS/GlcAT-S, was constructed as previously described [27]. HK-2 and HEK293 cells were cultured in Dulbecco’s Modified Eagle Medium (Cat#08456−65, Nacalai Tesque) supplemented with 10% fetal bovine serum (Cat#172012, Sigma-Aldrich) at 37 °C in 5% CO₂. Cells at ~80% confluence in 6-cm dishes were transfected with plasmids using Lipofectamine 3000 (Cat#L3000008, Thermo Fisher Scientific), following the manufacturer’s protocol, and cultured for 48 h. For glucose treatment, D-glucose was added at final concentrations of 5 mM or 25 mM at the time of transfection. For UDP-GlcA depletion, 4-methylumbelliferone (4-MU; Cat#M1381, Sigma-Aldrich) was added to a final concentration of 1 mM at 24 h post-transfection, and cells were incubated for an additional 24 h. Dimethyl sulfoxide (DMSO) alone was used as a vehicle control.

### Statistical analysis

Student’s *t*-test was used for comparisons between two groups. For comparisons among four groups, one-way ANOVA followed by Holm-Sidak post hoc test was applied by using OriginPro (OriginLab, Northampton, MA, USA). All source data for the graphs are included in S1 File.

## Results

### nsHNK-1 glycan expression is more abundant in female kidneys

**Fig 1:**
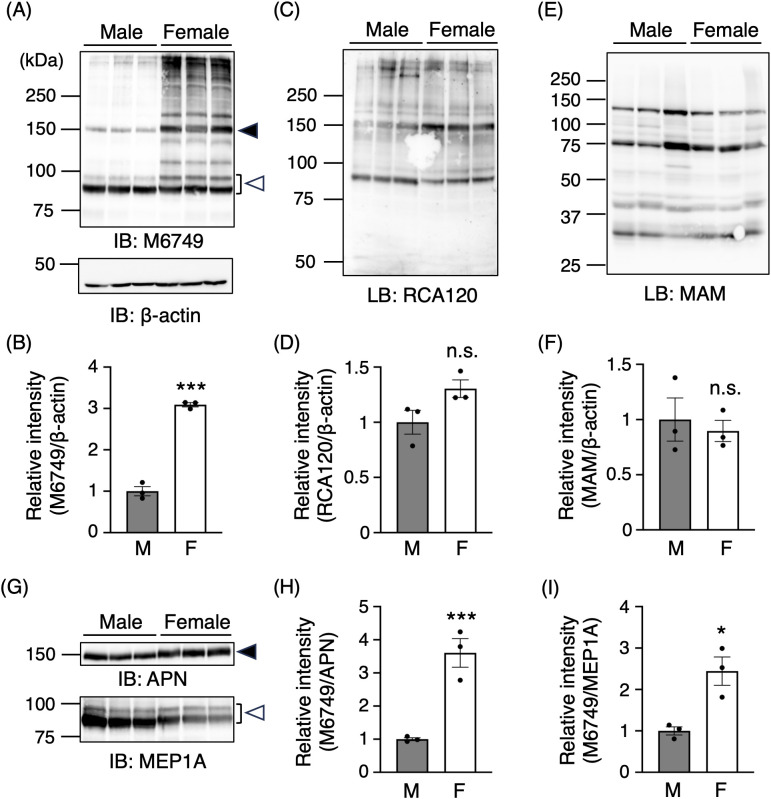
Nonsulfated HNK-1 glycan is highly expressed in female mouse kidney. **(A)** Renal membrane fractions from wild-type mouse kidneys were immunoblotted with the M6749 monoclonal antibody and anti-β-actin monoclonal antibody. β-actin was used as a loading control. All other blots in this figure were performed using the same loading amounts as in **(A)**. **(B)** Relative expression levels of nonsulfated HNK-1 glycans (M6749) were quantified based on the band intensities in **(A)**, normalized to β-actin. **(C, E)** Renal membrane fractions were blotted with RCA120 (C) and MAM (E) lectins. **(D, F)** Band signals in (C) and (E) were quantified relative to β-actin. **(G)** Immunoblots of renal membrane fractions using antibodies against APN and MEP1A. Bands marked by closed and open triangles correspond to the bands shown in **(A)**. (H, **I)** Expression levels of nonsulfated HNK-1 glycans associated with APN (H) and MEP1A (I) were quantified from the band intensities indicated by closed and open triangles. All graphs represent mean ± SEM (male, *n* = 3; female, *n* = 3). Statistical analysis was performed using Student’s *t*-test. *: *p* < 0.05, ***: *p* < 0.001, n.s.: not significant. “M” and “F” in figures indicate Male and Female, respectively.

Sex differences in renal expression of nsHNK-1 glycans were investigated by western blotting analysis of renal membrane fractions from male and female mice. nsHNK-1 glycan expression was approximately three-fold higher in female kidneys than in males (Fig 1A and 1B), revealing a significant sex difference in renal nsHNK-1 glycan expression. Since a similar trend was observed in another mouse strain (S1A Fig), the higher renal expression of nsHNK-1 glycans in female mice appears to be a conserved mechanism in mammals, at least in mice. The spatial distribution of nsHNK-1 glycan expression in the kidney was further characterized by performing immunofluorescence staining on renal sections from male and female mice (S1B Fig). Focusing on the cortical region, nsHNK-1 glycan was predominantly localized to proximal tubules in both sexes. However, in female kidneys, the expression extended more prominently into the outer layer of the cortical region. This distribution pattern closely resembled that of megalin, a well-established marker for proximal tubules that is expressed throughout the S1, S2, and S3 segments [28,29]. In contrast, MEP1A, another proximal tubule marker primarily localized to the S3 segment corresponding to proximal straight tubules [28,30], exhibited an expression pattern that overlapped with the male distribution of nsHNK-1 glycan but not with the expanded outer localization observed in females. These findings suggest that nsHNK-1 glycan expression may be relatively abundant in the S1 and S2 segments of the proximal tubules in the female mice.

To explore the possibility that increased expression levels of LacNAc structure may cause high expression of nsHNK-1 glycan in females, lectin blotting was conducted using RCA120, which binds terminal LacNAc structures (Fig 1C and 1D). Given that terminal sialic acid also modifies LacNAc and can potentially compete with GlcAT-S-mediated glucuronylation, blotting using sialic acid-specific lectins was additionally performed to assess sialylation levels (Fig 1E and 1F). A comparison between the sexes revealed that sialic acid levels were unaltered in both α2−3 and α2−6 sialylation (Fig 1E and 1F, S2C and S2D Fig). Likewise, LacNAc levels showed no significant sex difference (Fig 1C and D). Consistent with this finding, the mRNA level of *B4galt1*, which is involved in galactosylation of nonreducing terminus of N-glycan [31], did not show significant differences between sexes (S1E Fig). Furthermore, the mRNA levels of *Mgat4a*, *Mgat4b*, and *Mgat5*, which contribute to N-glycan branching [32], were also comparable between sexes, suggesting that the extent of glycan branching is unlikely to account for the high nsHNK-1 glycan expression in females. These findings suggest that glycan expression in female kidneys is not broadly increased compared with males. Rather, nsHNK-1 biosynthesis is selectively enhanced in female kidneys.

To determine whether high nsHNK-1 glycan expression in female kidneys is caused by increased levels of its known carrier proteins, western blotting was performed for APN and MEP1A, both previously identified as major carriers of nsHNK-1 glycans [4,9]. APN expression showed no significant sex difference, whereas MEP1A levels were slightly lower in females (Fig 1G). When normalized to the expression levels of their respective carrier proteins, nsHNK-1 glycan expression levels were approximately four-fold higher on APN and two-fold higher on MEP1A in female kidneys compared with male kidneys (Fig 1H and 1I). These results indicate that the high nsHNK-1 glycan expression in female kidneys is not attributable to increased expression of carrier proteins but reflects enhanced biosynthesis of nsHNK-1 glycan.

### Sex difference in renal nsHNK-1 expression is independent of GlcAT-S and GlcAT-P levels

**Fig 2:**
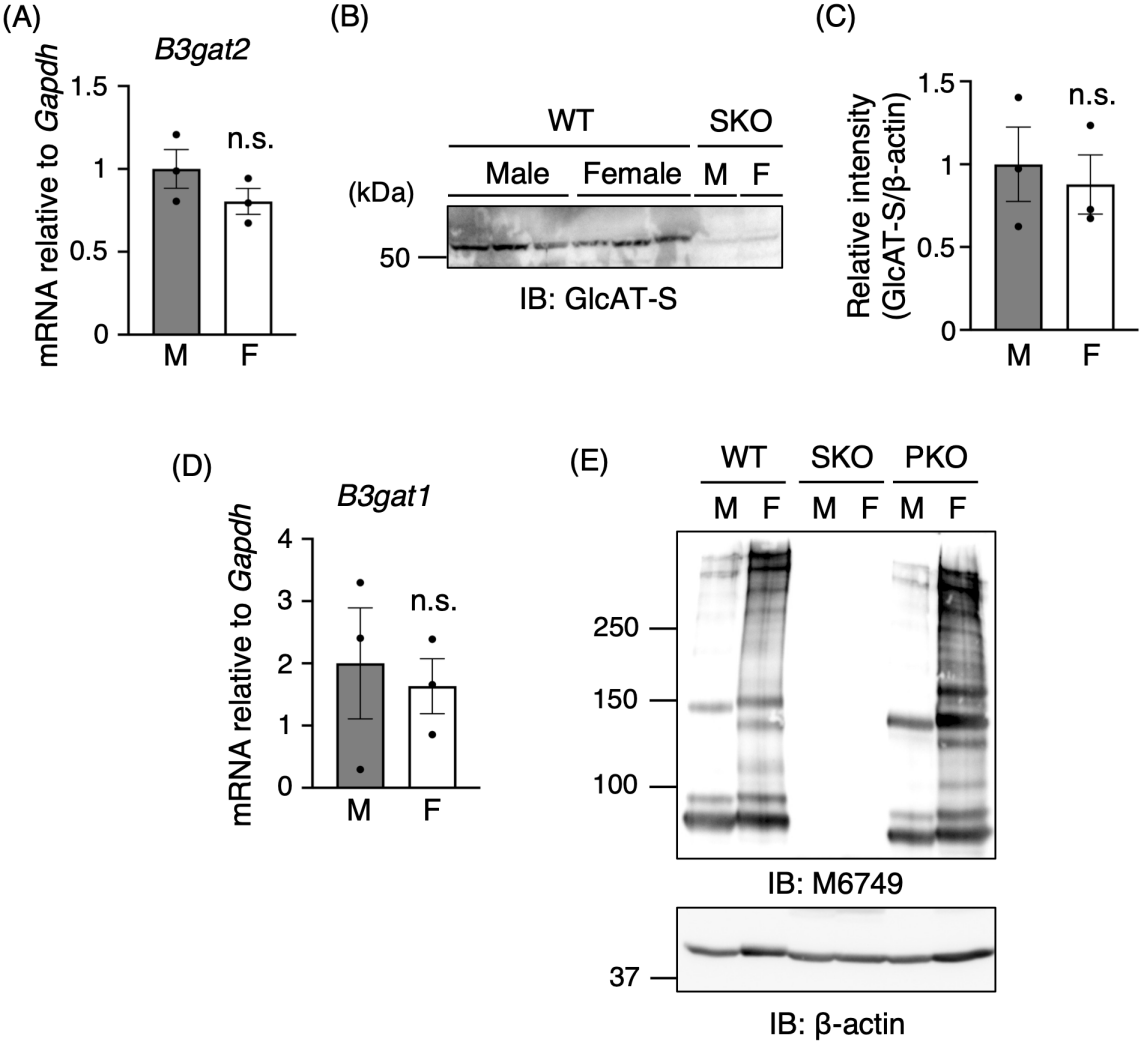
No sex difference in the expression of GlcAT-S and GlcAT-P. **(A)** *B3gat2* mRNA expression levels relative to *Gapdh* were determined by qPCR (male, *n* = 3; female, *n* = 3). **(B)** Renal membrane fractions prepared from wild-type (WT) and GlcAT-S gene knockout (SKO) mouse kidneys were immunoblotted with an anti-GlcAT-S polyclonal antibody. **(C)** GlcAT-S protein expression levels relative to β-actin were quantified based on the immunoblot in **(B)**. Band intensities of β-actin were identical to those in Fig 1A (male, *n* = 3; female, *n* = 3). **(D)** *B3gat1* mRNA expression levels relative to *Gapdh* were determined by qPCR (male, *n* = 3; female, *n* = 3). **(E)** Renal membrane fractions from WT, SKO, and GlcAT-P gene knockout (PKO) mouse kidneys were immunoblotted with the M6749 monoclonal antibody and anti-β-actin monoclonal antibody. All graphs show mean ± SEM. Statistical analysis was performed using Student’s *t*-test. n.s.: not significant. “M” and “F” in figures indicate Male and Female, respectively.

mRNA and protein levels of GlcAT-S (*B3gat2*) were examined to assess whether the abundant expression of nsHNK-1 glycans in female kidneys is caused by high expression of GlcAT-S. Quantitative PCR using cDNA from male and female mice kidneys exhibited no significant difference in *B3gat2* transcript levels between sexes (Fig 2A). Rather, a slight decrease was observed in females. Similarly, GlcAT-S protein levels showed no significant sex difference (Fig 2B and 2C), and the signal was completely absent in kidneys from SKO mice, confirming the specificity of the antibody (Fig 2B). These results suggest that the higher expression of nsHNK-1 glycans in female kidneys is not explained by GlcAT-S expression.

Although GlcAT-P is considered absent in the kidney, a previous proteomics study detected GlcAT-P peptides in rat collecting ducts [33]. Therefore, to address its potential involvement in the sex difference of nsHNK-1 glycan expression, the expression of *B3gat1* was assessed. No significant difference in *B3gat1* mRNA levels between sexes was observed (Fig 2D). Renal membrane fractions from both sexes of WT, SKO, and PKO mice were then compared to further evaluate the involvement of GlcAT-P in nsHNK-1 glycan biosynthesis (Fig 2E). In SKO mice, nsHNK-1 glycan expression was undetectable in both sexes, indicating that GlcAT-P is not sufficient to support nsHNK-1 glycan synthesis. Moreover, in PKO mice, nsHNK-1 glycan expression remained markedly higher in female kidneys compared with male kidneys, with an expression pattern resembling that observed for WT mice. These results collectively indicate that GlcAT-P does not contribute to the sex difference in renal nsHNK-1 glycan expression.

### Sex difference in renal UDP-GlcA levels implies higher utilization in female kidneys

GlcAT-S uses UDP-GlcA as the donor substrate to add glucuronic acid to the nonreducing terminus of LacNAc. However, UDP-GlcA is also used by UGTs, a family of enzymes that catalyze glucuronidation reactions [11]. Notably, previous RNA sequencing data from isolated mouse proximal tubules showed that Ugt1a2, a member of the UGT family, is expressed at significantly higher levels in females compared with males [19,20]. Additionally, urinary and blood levels of UDP-GlcA have been reported to differ between males and females [34]. These findings suggest that sex differences in UDP-GlcA availability contribute to the high expression of nsHNK-1 glycans in female kidneys.

**Fig 3:**
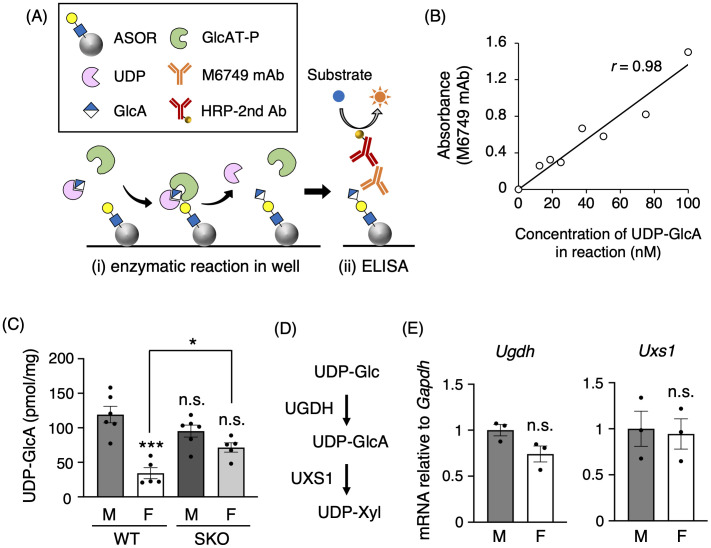
Low UDP-GlcA levels in female mouse kidneys. **(A)** Schematic overview of the ELISA-based assay developed to quantify UDP-GlcA levels. This method utilizes immobilized asialorosomucoid (ASOR) as a GlcA-accepting substrate and purified recombinant GlcAT-P to enzymatically transfer GlcA from UDP-GlcA to N-acetyllactosamine structures *in vitro*. The resulting nsHNK-1 glycans are detected using the M6749 monoclonal antibody, and UDP-GlcA levels are estimated based on HRP-derived signal intensity. **(B)** Validation of the method described in **(A)**. A standard curve was generated using serial dilutions of UDP-GlcA trisodium salt. **(C)** UDP-GlcA levels in kidneys from wild-type (WT) and GlcAT-S gene knockout (SKO) mice were measured using the ELISA-based assay described in **(A)** (WT male and SKO male, *n* = 6; WT female and SKO female, *n* = 5). Statistical analysis was performed using one-way ANOVA followed by Holm-Sidak post hoc test. *: *p* < 0.05, ***: *p* < 0.001. n.s.: not significant. The statistical test results shown in the graph are as follows: WT male vs. WT female, ***; WT male vs. SKO male, n.s.; WT male vs. SKO female, n.s.; WT female vs. SKO female, *. **(D)** Schematic of the UDP-GlcA biosynthetic pathway. Abbreviations: UDP-Glc, UDP-glucose; UDP-Xyl, UDP-xylose; UGDH, UDP-glucose dehydrogenase; UXS1, UDP-glucuronate decarboxylase 1. **(E)** *Ugdh* and *Uxs1* mRNA expression levels relative to *Gapdh* in WT mouse kidneys were determined by qPCR (male, *n* = 3; female, *n* = 3). Statistical analysis was performed using Student’s *t*-tes*t*. n.s.: not significant. All graphs show mean ± SEM. “M” and “F” in figures indicate Male and Female, respectively.

Quantifying UDP-GlcA levels was achieved by developing a simple and reproducible ELISA-based assay based on a previous report with slight modifications (Fig 3A) [23]. In this method, ASOR was immobilized on a microplate as the substrate for GlcA modification. UDP-GlcA was then added with purified recombinant GlcAT-P, which was expressed in *Escherichia coli*. The reaction was performed at 37 °C, allowing GlcAT-P to transfer glucuronic acid from UDP-GlcA to LacNAc, thereby generating nsHNK-1 structures *in vitro*. The resulting nsHNK-1 glycans were detected with the M6749 mAb, and UDP-GlcA levels were estimated based on the HRP-dependent signal intensity. The feasibility of this assay was validated by generating a standard curve using serial dilutions of the UDP-GlcA trisodium salt. A strong linear correlation was observed between the UDP-GlcA concentration and absorbance, confirming that the assay reliably quantifies UDP-GlcA levels (Fig 3B, S2A Fig). Note that the absorbance remained unchanged upon addition of kidney extracts to the assay, indicating that interfering factors derived from kidney tissue are negligible in this measurement system (S2B Fig).

Using this assay, renal UDP-GlcA levels were measured (Fig 3C). In WT mice, UDP-GlcA levels were significantly lower in females than in males, suggesting greater use of this compound in female kidneys. This sex difference was abolished in SKO mice, indicating that GlcAT-S-mediated consumption contributes to the low UDP-GlcA levels in females. Notably, UDP-GlcA levels were significantly high in SKO females compared with WT females, and although these levels in SKO females tended to be slightly lower than those in WT males, the difference was not significant. Additionally, to explore whether sex differences in UDP-GlcA levels arise from the biosynthetic pathway of UDP-GlcA, the expression of UDP-glucose dehydrogenase and UDP-glucuronate decarboxylase 1, which are key enzymes involved in UDP-GlcA metabolism (Fig 3D) [10,35], was examined. No significant differences in their mRNA expression were observed between males and females (Fig 3E). These findings suggest that the biosynthetic level of UDP-GlcA does not differ substantially between sexes, supporting the notion that UDP-GlcA is actively consumed in a GlcAT-S-dependent manner in female kidneys.

### UDP-GlcA availability mediates nsHNK-1 glycan expression

**Fig 4:**
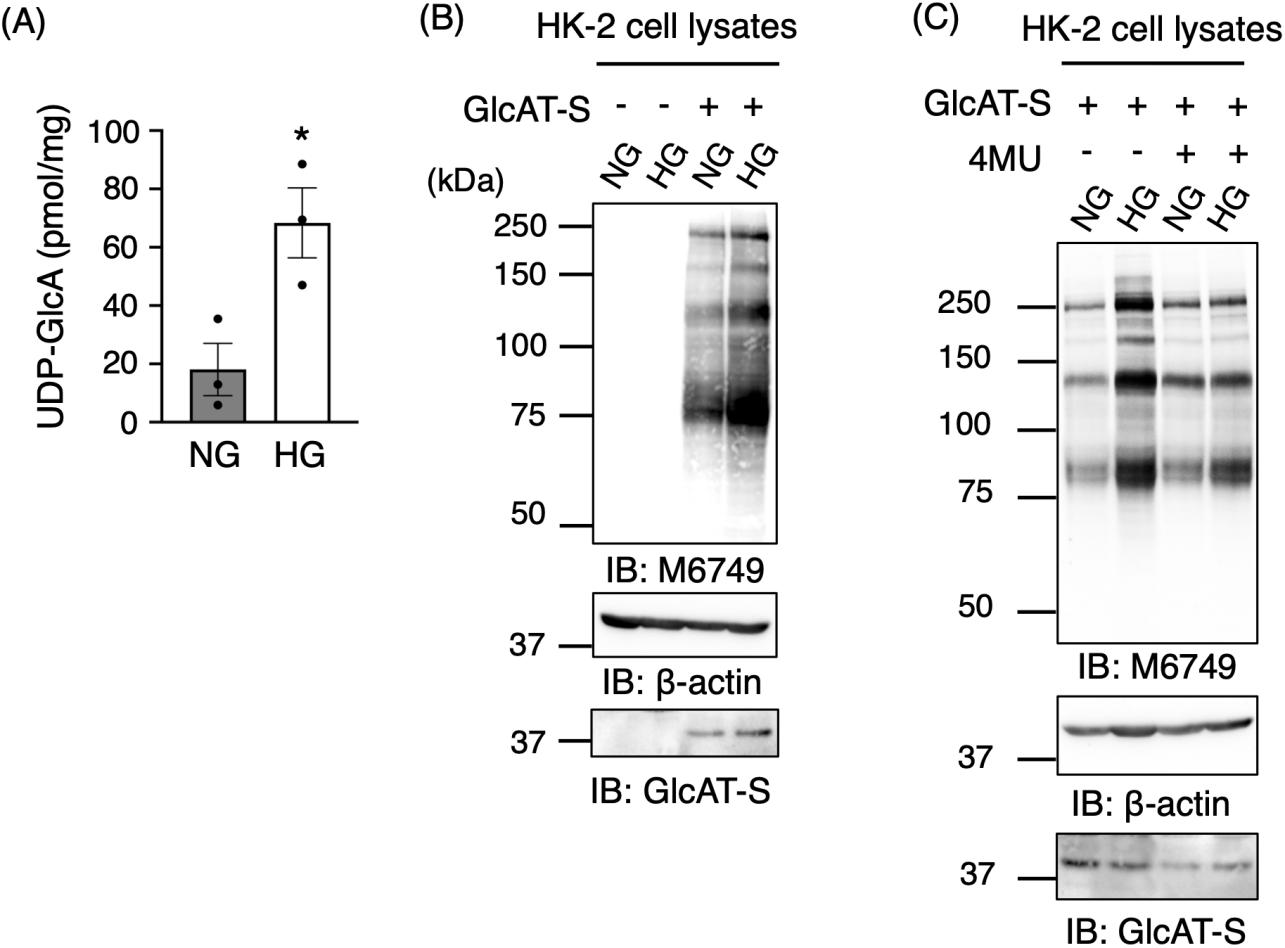
High UDP-GlcA levels increase nsHNK-1 glycan expression. **(A)** HK-2 cells were cultured for 48 hours in medium containing either 5 mM glucose (normal glucose, NG) or 25 mM glucose (high glucose, HG). UDP-GlcA levels were then quantified using the ELISA-based assay described in Fig 3A. Graphs represent mean ± SEM (NG, *n* = 3; HG, *n* = 3). Statistical analysis was performed using Student’s *t*-test. *: *p* < 0.05. **(B)** Cells were transfected with or without GlcAT-S and subsequently cultured under NG or HG conditions for 48 **h.** Cell lysates were immunoblotted with the M6749 monoclonal antibody, anti-β-actin monoclonal antibody, and anti-GlcAT-S polyclonal antibody. **(C)** GlcAT-S-expressing cells were incubated under NG and HG conditions. After 24 h of incubation, 4-methylumbelliferone (4-MU) was added to the culture medium at a final concentration of 1 mM. Cell lysates were then immunoblotted with the M6749 monoclonal antibody, anti-β-actin monoclonal antibody, and anti-GlcAT-S polyclonal antibody.

The influence of intracellular UDP-GlcA levels on nsHNK-1 glycan expression was investigated using an *in vitro* approach. Increasing the glucose concentration in culture media increases UDP-GlcA levels through enhanced flux in the nucleotide sugar biosynthetic pathway [36]. Consistently, culturing HK-2 cells under high-glucose (HG, 25 mM) conditions caused a significant increase in intracellular UDP-GlcA compared with normal glucose (NG, 5 mM) conditions (Fig 4A). HK-2 cells essentially lack endogenous GlcAT-S. Thus, GlcAT-S was transfected into the cells to allow nsHNK-1 glycan synthesis. Under these conditions, a significant increase in nsHNK-1 glycan expression was observed in HG-cultured cells relative to NG (Fig 4B). This glucose-dependent upregulation of nsHNK-1 glycans was also reproduced in HEK293 cells (S3 Fig), suggesting that this regulatory mechanism is preserved across distinct kidney-derived cell types. 4-MU was added to the culture media to assess whether this effect was dependent on UDP-GlcA availability. 4-MU acts as a glucuronidation substrate, reducing intracellular UDP-GlcA levels through its consumption by UGT enzymes [37]. In the presence of 4-MU, the difference in nsHNK-1 glycan expression between NG and HG conditions was no longer detectable (Fig 4C). Collectively, these results demonstrate that glucose-induced increases in UDP-GlcA levels are sufficient to drive nsHNK-1 glycan expression, underscoring intracellular UDP-GlcA availability as a key metabolic determinant of nsHNK-1 glycan biosynthesis in the kidney.

## Discussion

The expression of nsHNK-1 glycan in the kidney has been extensively studied in previous research, particularly with regard to its localization and age-related increase [4,9]. However, potential sex differences in nsHNK-1 glycan expression have not been investigated previously. In this study, a clear sex-dependent difference in renal nsHNK-1 glycan expression was identified, with significantly higher expression levels observed in female mouse kidneys (Fig 1A, S1A Fig). Notably, this difference occurred without corresponding changes in GlcAT-S expression at either the mRNA or protein level (Fig 2A–2C). Sex-biased protein expression is generally attributed to hormonal regulation, which also affects the expression of glycosyltransferases. Sex hormones have been shown to modulate the expression of various glycosyltransferases [14]. As an example of hormone-induced alterations in nonreducing terminal modifications, estrogen treatment increases the expression of the sialyltransferase ST6Gal1 in ovariectomized mice, resulting in enhanced IgG sialylation [38]. Estrogen also modulates IgG galactosylation levels in both men and women [39]. These effects are typically mediated through hormone receptors binding to gene promoters or via transcriptional regulation involving microRNAs [14]. However, GlcAT-S expression does not appear to be hormonally regulated. RNA sequencing of isolated proximal tubules has shown no significant sex differences in *B3gat2* transcript levels [19,20], and our data similarly showed no sex-specific variation in GlcAT-S expression (Fig 2A–2C). These findings indicate that the observed sex difference in nsHNK-1 glycan expression is unlikely to result from direct hormonal regulation of GlcAT-S gene expression. Another possible pathway affecting nsHNK-1 glycan expression is its degradation. The low nsHNK-1 expression in male kidneys could hypothetically result from enhanced degradation of the nsHNK-1-carrier proteins; however, this possibility can be ruled out, as there were no sex differences in the expression levels of APN or MEP1A (Fig 1G). Although one might consider the selective degradation of nsHNK-1 glycans at the plasma membrane, to date, no reports have described such a degradation pathway. Given nsHNK-1 degradation at the plasma membrane occurred, the reactivity of M6749 mAb toward MEP1A would be expected to decrease in male kidneys more markedly than observed. However, the observed differences are minimal (Fig 1A, open triangle), and thus, this possibility also appears unlikely. Collectively, these findings suggest that degradation pathways are not a major determinant of nsHNK-1 glycan expression.

Prompted by the female-predominant expression of Ugt1a2 in proximal tubules [19,20], renal UDP-GlcA levels were quantified to explore potential sex differences in its metabolism. Using our established method, we found that UDP-GlcA concentrations were significantly lower in female WT mice than in male mice (Fig 3C), suggesting increased utilization of this compound in female kidneys. This observation may reflect higher GlcAT-S enzymatic activity. However, direct quantification of GlcAT-S activity in kidney tissue remains technically challenging, leaving the question of sex-dependent activity unresolved. Moreover, the molecular mechanism underlying the putative increase in GlcAT-S activity in females remains poorly understood. Although posttranslational modifications affecting GlcAT-S activity may be involved, immunoblotting analysis revealed no apparent sex difference in GlcAT-S molecular weight (Fig 2B). Overall, elucidating the regulatory mechanisms governing potential sex differences in GlcAT-S activity will be an important objective for future studies.

Immunohistochemical analysis suggested that nsHNK-1 glycan expression is enriched in the outer cortical region of female mice compared to male mice, implicating increased expression within the S1 and S2 segments of proximal tubules (S1B Fig). Notably, nsHNK-1 glycan expression was also abundant on MEP1A (Fig 1G and 1I), raising the possibility that S3 segments may also contribute to the high nsHNK-1 glycan expression. Furthermore, APN is expressed throughout all segments of proximal tubules [30], suggesting that abundant nsHNK-1 glycan expression on APN accounts for the high expression pattern in S1 and S2. However, the identities of renal nsHNK-1-carrier proteins remain largely uncharacterized. Laminin-1, one of the extracellular matrix proteins, is an nsHNK-1-carrier protein [40], but broadly distributed throughout the nephron, including glomeruli and Henle’s loop [41]. These indicate that other, yet unidentified, proteins may account for the segment-specific enrichment of nsHNK-1 glycan seen in females. Importantly, the expression levels of known carrier proteins such as MEP1A and APN do not differ significantly between sexes, indicating that the observed sex-dependent increase in nsHNK-1 glycan expression arises from differential glycan modification rather than changes in carrier abundance. This result indicates that variations in UDP-GlcA availability modulate the degree of nsHNK-1 glycan modification of these carrier proteins, enabling flexible and dynamic regulation of their function without changes in carrier protein abundance. Future studies employing spatial transcriptomics or segment-specific proteomics will be essential in identifying critical carrier proteins and elucidating the regulatory network underlying sex-specific glycosylation patterns.

Sex differences in renal function are well documented. Men have a higher risk of acute kidney injury and generally experience more rapid progression to end-stage chronic kidney disease than women [42,43]. The most common cause of acute kidney injury is ischemia-reperfusion injury, which leads to cell death primarily in proximal tubular epithelial cells [44]. Similarly, proximal tubule injury is a central feature of chronic kidney disease, with both the severity and frequency of such damage influencing disease progression [45]. These observations underscore the importance of sex-specific functional differences in the proximal tubules, consistent with previous reports of distinct gene expression patterns in this nephron segment between the sexes [19,20]. Our study provides the first evidence of sex-dependent variation in renal nsHNK-1 glycan expression, suggesting a potential protective role for nsHNK-1 glycans in the proximal tubules of female kidneys. This postulate suggests that nsHNK-1 glycan may contribute to sex-specific renal physiology and resilience against injury. A comprehensive investigation of the functional roles of nsHNK-1 glycan within the kidney, particularly concerning sex differences in health and disease, represents an important future research topic. Such studies may provide deeper insight into the mechanisms underlying sex differences in renal function and contribute to the development of more effective strategies for treating kidney disease.

## Supporting information

S1 Fig Sex differences in the regional expression of renal nsHNK-1 glycan and glycosyltransferase mRNA levels. (A) Renal membrane fractions from BALB/c mouse kidneys were immunoblotted with the M6749 monoclonal antibody and anti-β-actin monoclonal antibody. Note that BALB/c mice were used only for this experiment. (B) Cortical sections from wild-type mouse kidneys were immunostained with anti-megalin polyclonal antibody and anti-MEP1A polyclonal antibody. Megalin was localized to proximal tubules throughout the S1, S2, and S3 segments, while MEP1A was restricted to the S3 segment, corresponding to the proximal straight tubules (left two panels). Sections from wild-type male and female mouse kidneys were also immunostained with the M6749 monoclonal antibody (right two panels). In male kidneys, nsHNK-1 glycan expression was primarily observed in the S3 segment, as indicated by its similar distribution to MEP1A. In contrast, female kidneys showed additional nsHNK-1 glycan expression in the outer cortical region, suggesting that nsHNK-1 is additionally expressed in the S1 and S2 segments, which correspond to proximal convoluted tubules. (C) Renal membrane fractions were blotted with SiaFind α2–6. (D) Signals were quantified relative to β-actin based on the band intensities in Fig 1A. (E) mRNA expression levels of *B4galt1*, *Mgat4a*, *Mgat4b*, and *Mgat5* relative to *Gapdh* were determined by qPCR (male, *n* = 3; female, *n* = 3). Statistical analysis was performed using Student’s *t*-test. n.s.: not significant. All graphs show mean ± SEM. “M” and “F” in figures indicate Male and Female, respectively.
(PDF)

S2 Fig Validation of ELISA for UDP-GlcA quantification. (A) Standard curves were generated using serial dilutions of UDP-GlcA trisodium salt. This experiment was independently performed three times, and the black line is shown in Fig 3B as a representative standard curve. (B) The absorbance at 37.5 nM UDP-GlcA was measured in the presence or absence of kidney tissue extracts (*n* = 3). Statistical analysis was performed using Student’s *t*-test. n.s.: not significant. Graphs show mean ± SEM.
(PDF)

S3 Fig High glucose condition elevates nsHNK-1 glycan expression. HEK293 cells were transfected with or without GlcAT-S and subsequently cultured for 48 h under either 5 mM (normal glucose, NG) or 25 mM (high glucose, HG) glucose conditions. Cell lysates were immunoblotted with the M6749 monoclonal antibody, anti-β-actin monoclonal antibody, and anti-GlcAT-S polyclonal antibody.
(PDF)

S4 Fig Western blot images including molecular size markers. Dashed lines indicate the regions used in Figures. White and black arrows indicate the molecular weight marker lanes.
(PDF)

S1 File Raw data used to generate the graphs shown in the figures. (XLSX)
