# Supplementary figures and images for: UDP-glucuronic acid availability underlies sex difference in renal expression of nonsulfated Human Natural Killer-1 (HNK-1) glycans
Source: PLoS One. 2025 Nov 13;20(11):e0335730. doi: 10.1371/journal.pone.0335730 (PMC12614588; doi:10.1371/journal.pone.0335730)

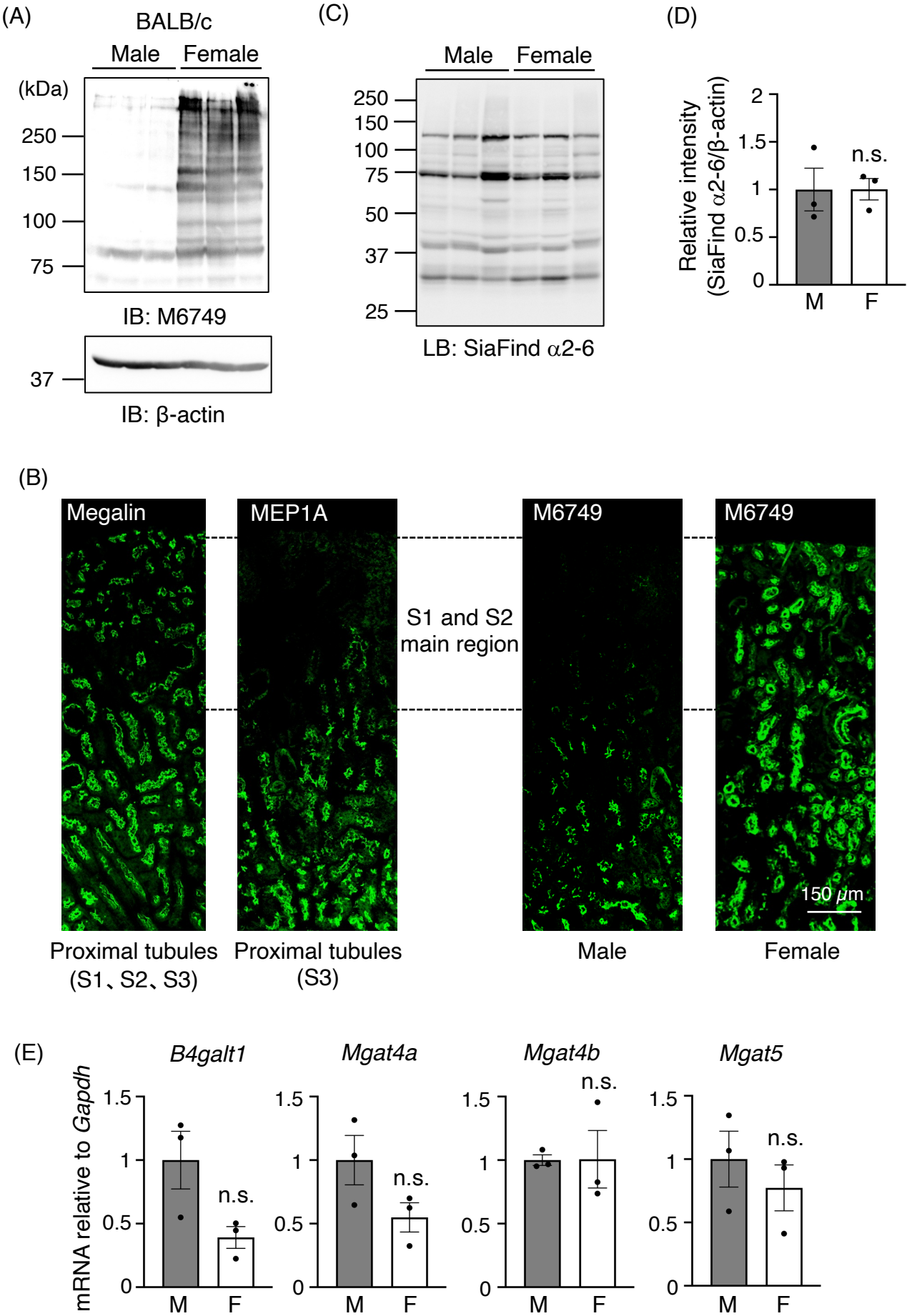

S1 Fig

Supplement: S1 Fig — (A) Renal membrane fractions from BALB/c mouse kidneys were immunoblotted with the M6749 monoclonal antibody and anti-β-actin monoclonal antibody. Note that BALB/c mice were used only for this experiment. (B) Cortical sections from wild-type mouse kidneys were immunostained with anti-megalin polyclonal antibody and anti-MEP1A polyclonal antibody. Megalin was localized to proximal tubules throughout the S1, S2, and S3 segments, while MEP1A was restricted to the S3 segment, corresponding to the proximal straight tubules (left two panels). Sections from wild-type male and female mouse kidneys were also immunostained with the M6749 monoclonal antibody (right two panels). In male kidneys, nsHNK-1 glycan expression was primarily observed in the S3 segment, as indicated by its similar distribution to MEP1A. In contrast, female kidneys showed additional nsHNK-1 glycan expression in the outer cortical region, suggesting that nsHNK-1 is additionally expressed in the S1 and S2 segments, which correspond to proximal convoluted tubules. (C) Renal membrane fractions were blotted with SiaFind α2–6. (D) Signals were quantified relative to β-actin based on the band intensities in Fig 1A. (E) mRNA expression levels of B4galt1, Mgat4a, Mgat4b, and Mgat5 relative to Gapdh were determined by qPCR (male, n = 3; female, n = 3). Statistical analysis was performed using Student’s t-test. n.s.: not significant. All graphs show mean ± SEM. “M” and “F” in figures indicate Male and Female, respectively. (PDF) [file pone.0335730.s001.pdf]

(A)

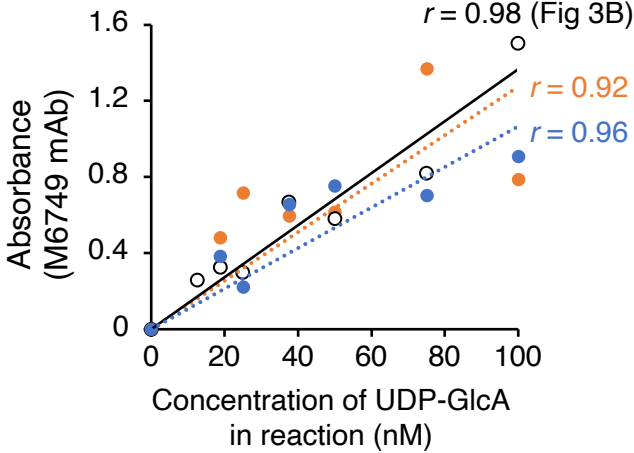

(B)

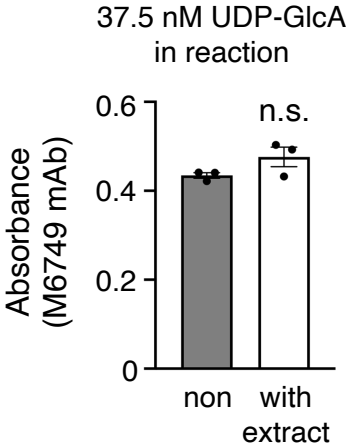

S2 Fig

Supplement: S2 Fig — (A) Standard curves were generated using serial dilutions of UDP-GlcA trisodium salt. This experiment was independently performed three times, and the black line is shown in Fig 3B as a representative standard curve. (B) The absorbance at 37.5 nM UDP-GlcA was measured in the presence or absence of kidney tissue extracts (n = 3). Statistical analysis was performed using Student’s t-test. n.s.: not significant. Graphs show mean ± SEM. (PDF) [file pone.0335730.s002.pdf]

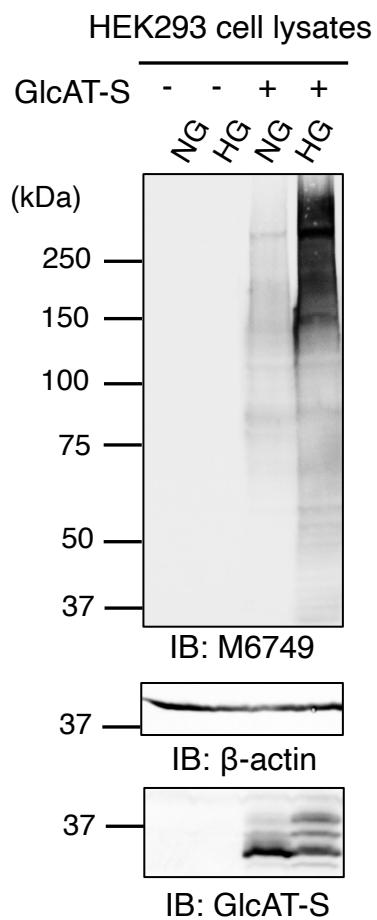

S3 Fig

Supplement: S3 Fig — HEK293 cells were transfected with or without GlcAT-S and subsequently cultured for 48 h under either 5 mM (normal glucose, NG) or 25 mM (high glucose, HG) glucose conditions. Cell lysates were immunoblotted with the M6749 monoclonal antibody, anti-β-actin monoclonal antibody, and anti-GlcAT-S polyclonal antibody. (PDF) [file pone.0335730.s003.pdf]
