# Supplementary material for: UDP-glucuronic acid availability underlies sex difference in renal expression of nonsulfated Human Natural Killer-1 (HNK-1) glycans
Source: PLoS One. 2025 Nov 13;20(11):e0335730. doi: 10.1371/journal.pone.0335730 (PMC12614588; doi:10.1371/journal.pone.0335730)
Supplement: S4 Fig — Dashed lines indicate the regions used in Figures. White and black arrows indicate the molecular weight marker lanes. (PDF) [file pone.0335730.s004.pdf]

Fig 1A, upper

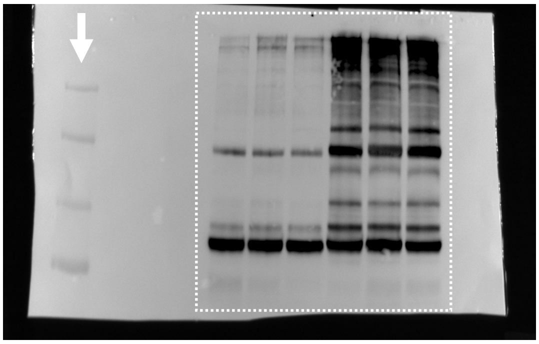

Fig 1C

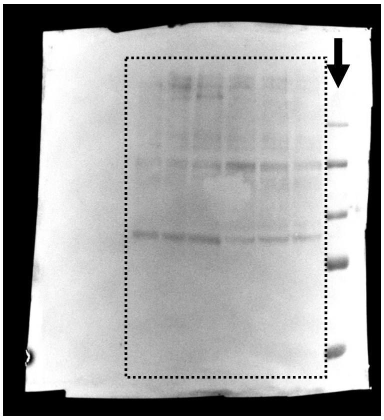

Fig 1E

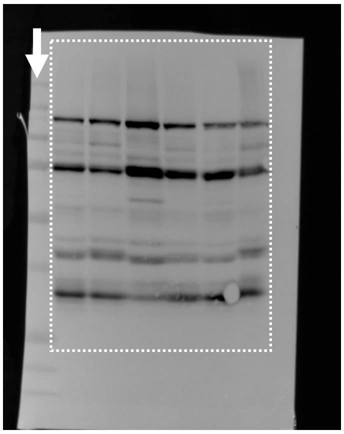

Fig 1A, lower

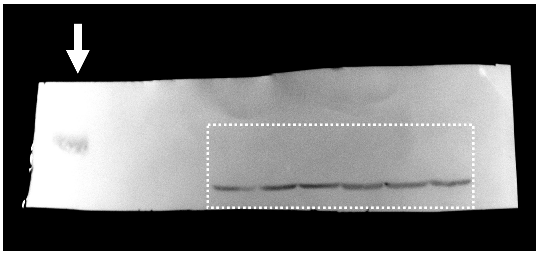

Fig 2B

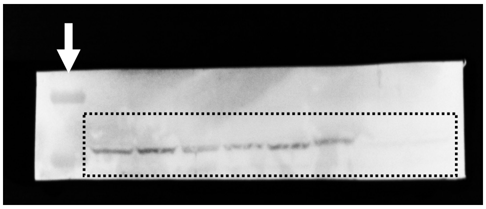

Fig 1G, upper

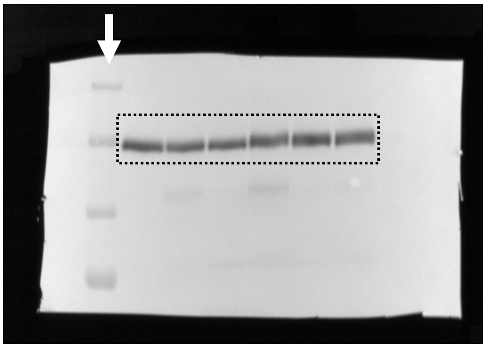

Fig 2E, upper

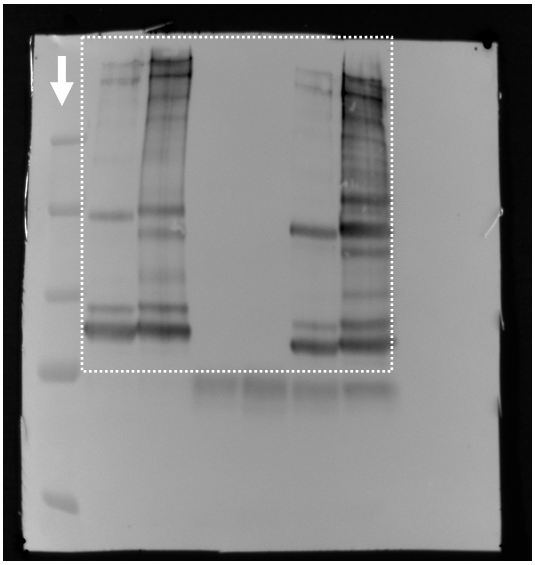

Fig 1G, lower

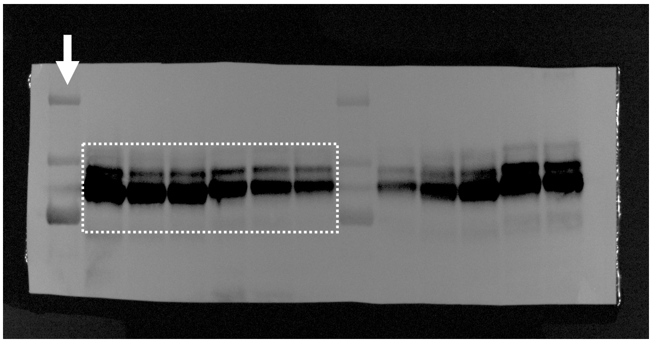

Fig 2E, lower

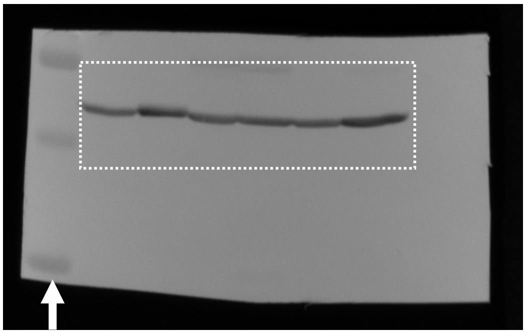

(continued to the next page)

S4 Fig

Fig 4B, upper

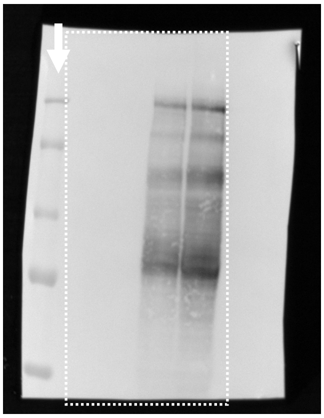

Fig 4B, middle

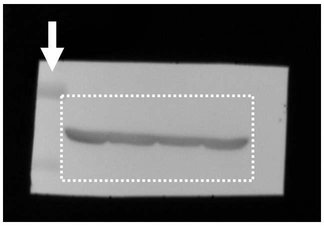

Fig 4B, lower

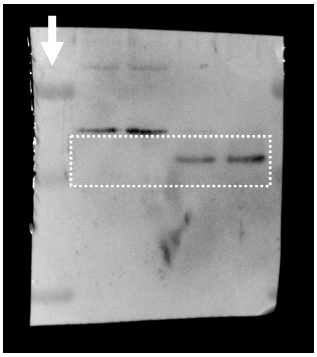

S1A Fig, upper

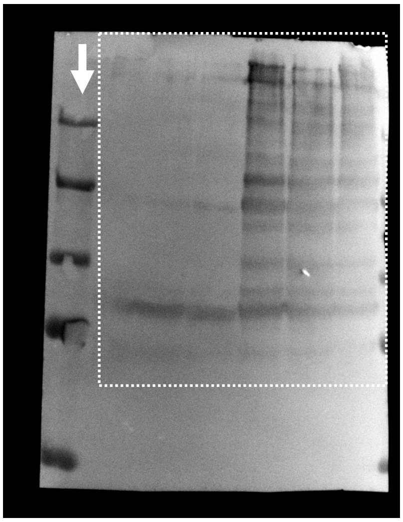

S1A Fig, lower

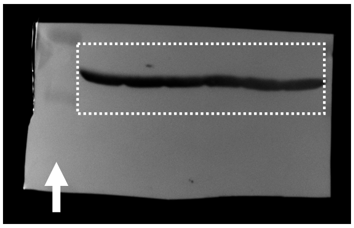

Fig 4C, upper

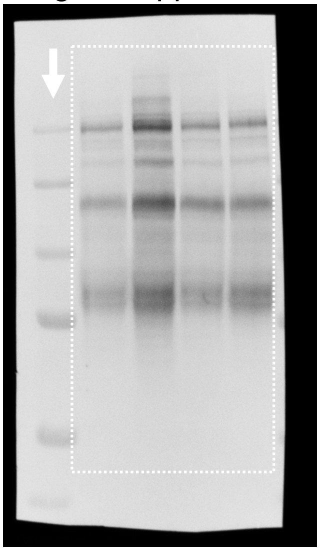

Fig 4C, middle

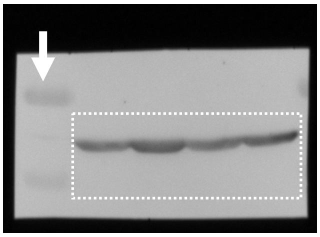

Fig 4C, lower

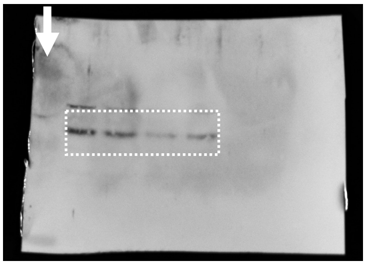

S1B Fig

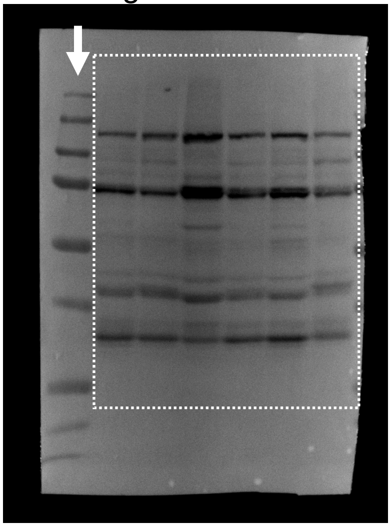

S3 Fig, upper

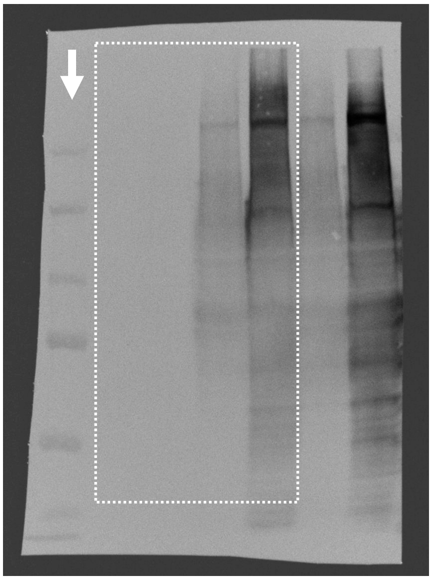

S3 Fig, middle

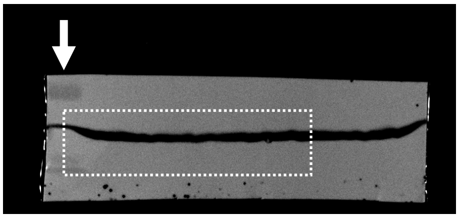

S3 Fig, lower

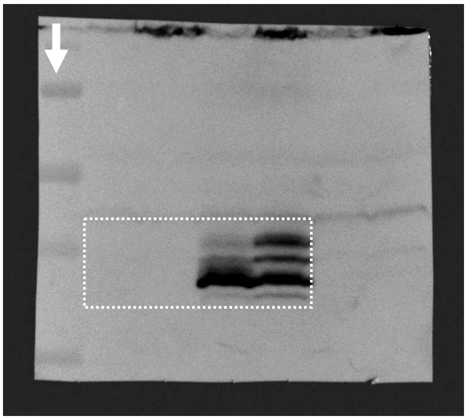

S4 Fig
